# Draft Genome Sequence of Enterococcus faecalis Strain CECT7121, a Corn Silage Isolate with Antibacterial Activity against Gram-Positive Pathogens

**DOI:** 10.1128/MRA.00245-19

**Published:** 2019-05-16

**Authors:** Gastón Delpech, Elvira M. Hebert, Mónica Sparo, Lucila Saavedra

**Affiliations:** a Microbiología Clínica, Medicina, Universidad Nacional del Centro de la Provincia de Buenos Aires, Olavarría, Argentina; b Laboratorio de Genética y Biología Molecular, Centro de Referencia para Lactobacilos (CERELA-CONICET), San Miguel de Tucumán, Argentina; Indiana University, Bloomington

## Abstract

Enterococcus faecalis CECT7121 is a corn silage probiotic bacterium that shows antibacterial activity against Gram-positive pathogens from different origins. Its genome sequence is 2.9 Mb long with a G+C content of 37.3%. Genome annotation identified three bacteriocin gene clusters in the genome.

## Abstract (precis)

Enterococcus faecalis CECT7121 is a corn silage probiotic bacterium that shows antibacterial activity against Gram-positive pathogens from different origins. Its genome sequence is 2.9 Mb long with a G+C content of 37.3%.

## ANNOUNCEMENT

Enterococcus faecalis CECT7121 (formerly MR99) is a probiotic strain isolated from Argentinean corn silage that has bactericidal activity against Gram-positive bacteria (1, 2). E. faecalis CECT7121 was grown in brain heart infusion broth (Laboratorios Britania, Buenos Aires, Argentina) at 35 ± 2°C for 18 h (2), and genomic DNA isolation was performed as described by Brown et al. (3).

Genome libraries for DNA sequencing were constructed using a TruSeq DNA PCR-free library preparation kit (Illumina, Inc.) with an insert size of 350 bp. The sequencing process was carried out at Macrogen, Inc. (Seoul, Republic of Korea) using an Illumina HiSeq platform. The paired-end library generated 17,520,654 reads (101-bp paired end). The quality of the raw data was checked with FastQC (4); Trimmomatic was used both to remove low-quality sequences (Q ≤ 20) and to trim adapters (5). Reads were assembled *de novo* using SPAdes 3.12.0 (6) and annotated with the NCBI Prokaryotic Genome Annotation Pipeline (7). The draft genome sequence of E. faecalis CECT7121 has a length of 2,901,597 bp with a G+C content of 37.3% and was assembled into 17 contigs with an *N*_50_ value of 386,449 bp. This genome contains 2,821 coding genes and 69 RNAs.

An *in silico* genomic screening of biotechnological properties evidenced that the E. faecalis CECT7121 genome contains genes involved in bacteriocin production. The bacteriocin-mining software tool BAGEL4 identified 3 areas of interest (AOI) in contigs 12, 9, and 7, corresponding to potential bacteriocin genes in the genome of strain CECT7121. BLAST analysis determined that contig 12.7.AOI_01 contains 2 open reading frames (ORFs) encoding putative enterocin P (E value, 2 × 10^−29^) and its immunity protein (O30435_ENTFC; E value, 3 × 10^−27^). Sequence analysis of this gene cluster revealed the same genetic organization previously described (8). Contig 9.9.AOI_01 revealed an ORF related to enterocin SE-K4 gene cluster. The deduced amino acid sequence of this putative bacteriocin gene exhibited a high degree of identity with a hypothetical protein widely distributed among E. faecalis strains. However, a 5-amino acid deletion at the C-terminal domain was observed when it was compared to enterocin SE-K4 (*ent* SE-K4), first described in E. faecalis K4 (UniProtKB identifier Q8GR39_ENTFL). An *entA*_im_ gene, encoding a putative immunity protein from the enterocin A cluster (E value, 1 × 10^−30^), was found immediately downstream of the bacteriocin gene. In addition, a putative promoter sequence was predicted upstream of the bacteriocin gene. Nevertheless, the presence of two putative rho-independent terminators, just before and after these two gene clusters, suggests that they might not be functional. Contig 7.1.AOI_01 exhibits putative enterocin P and enterocin SE-K4 clusters as well. A putative enterocin P gene (E value, 2 × 10^−25^) and its immunity protein (ORF 00018; E value, 3 × 10^−26^; UniProtKB identifier O30435_ENTFC) are located downstream of a putative *ent* SE-K4 (E value, 3 × 10^−51^) and ORF 00015 (with no function determined). No putative promoter sequences were predicted upstream of these genes. Rho-independent terminators were predicted downstream of ORF 00015 and ORF 00018.

Based on the above-described analyses, we might speculate that the functional bacteriocin gene clusters are those described in contigs 12 (enterocin P) and 7 (enterocin SE-K4).

### Data availability.

This whole-genome shotgun project has been deposited at DDBJ/ENA/GenBank under the accession number SIJP00000000. The version described in this paper is the first version, SIJP01000000. Raw data are available under SRA accession number PRJNA523127.
